# An Integrated Optogenetic and Bioelectronic Platform for Regulating Cardiomyocyte Function

**DOI:** 10.1002/advs.202402236

**Published:** 2024-07-25

**Authors:** Olurotimi A. Bolonduro, Zijing Chen, Corey P. Fucetola, Yan‐Ru Lai, Megan Cote, Rofiat O. Kajola, Akshita A. Rao, Haitao Liu, Emmanuel S. Tzanakakis, Brian P. Timko

**Affiliations:** ^1^ Department of Biomedical Engineering Tufts University Medford MA 02155 USA; ^2^ Department of Chemical and Biological Engineering Tufts University Medford MA 02155 USA; ^3^ General Surgery Department Children's Hospital Zhejiang University School of Medicine, National Clinical Research Center for Children's Health Hangzhou 310052 China; ^4^ Cell, Molecular and Developmental Biology Graduate School of Biomedical Sciences Tufts University Boston MA 02111 USA; ^5^ Clinical and Translational Science Institute Tufts Medical Center Boston MA 02111 USA

**Keywords:** bioelectronics, cardiomyocytes, electrophysiology, multi‐electrode arrays, optogenetics

## Abstract

Bioelectronic medicine is emerging as a powerful approach for restoring lost endogenous functions and addressing life‐altering maladies such as cardiac disorders. Systems that incorporate both modulation of cellular function and recording capabilities can enhance the utility of these approaches and their customization to the needs of each patient. Here we report an integrated optogenetic and bioelectronic platform for stable and long‐term stimulation and monitoring of cardiomyocyte function in vitro. Optical inputs are achieved through the expression of a photoactivatable adenylyl cyclase, that when irradiated with blue light causes a dose‐dependent and time‐limited increase in the secondary messenger cyclic adenosine monophosphate with subsequent rise in autonomous cardiomyocyte beating rate. Bioelectronic readouts are obtained through a multi‐electrode array that measures real‐time electrophysiological responses at 32 spatially‐distinct locations. Irradiation at 27 µW mm^−2^ results in a 14% elevation of the beating rate within 20–25 min, which remains stable for at least 2 h. The beating rate can be cycled through “on” and “off” light states, and its magnitude is a monotonic function of irradiation intensity. The integrated platform can be extended to stretchable and flexible substrates, and can open new avenues in bioelectronic medicine, including closed‐loop systems for cardiac regulation and intervention, for example, in the context of arrythmias.

## Graphical abstract

This study reports an integrated platform for simultaneous modulation and monitoring of cardiomyocyte function in vitro. Optical inputs are achieved through expression of a photoactivatable adenylyl cyclase, that when irradiated causes a reversible increase in beating rate. Bioelectronic outputs are achieved with a multi‐electrode array, which can record the beating rate and wavefront propagation characteristics with high spatiotemporal resolution.

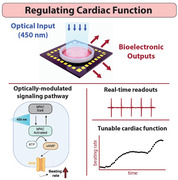

## 1 Introduction

Bioelectronic medicine (BM) is an emerging field aimed at treating pathologies with devices that modulate the response of electrically‐active cells. As a distinct departure from conventional pharmacological interventions, these therapies can be localized to a particular region of the body and provide stimuli that are personalized to the patient, potentially improving clinical outcomes.^[^ ^1^, ^2^ ^]^ Platforms that can both control and monitor tissue functions may offer further advantages through closed‐loop feedback for intervention in the event of an acute anomaly, or adapt the therapy should baseline activity change over time. BM could be especially transformative in cardiology, where devices implanted onto the vagus nerve or the heart could address indications such as cardiac arrythmia, atrial fibrillation and cardiomyopathy.^[^ ^3^, ^4^, ^5^ ^]^ These devices, however, will require seamless integration with the surrounding tissue and minimal – if any – inflammation at chronic time points.

Bioelectronic recording elements such as multi‐electrode arrays (MEAs) and field‐effect transistors are especially attractive in BM.^[^ ^6^, ^7^ ^]^ These devices couple with electrically‐active cells to provide continuous, real‐time readouts of spikes that correlate with action potentials. They play an important role in cardiac tissue engineering, as biopolymer scaffolds with MEAs or multiplexed transistors have been integrated with 3D tissue constructs to interrogate tissue development and electrical synchrony.^[^ ^8^, ^9^, ^10^, ^11^ ^]^ These systems cause minimal disruption to the cell membrane and do not elicit pronounced immune response making them uniquely suitable for long‐term tissue integration.^[^ ^12^ ^]^ Bioelectronic scaffolds within cardiac organoids have tracked organogenesis for 35 days,^[^ ^13^ ^]^ while similar devices implanted in a rodent model provided readouts from the same set of cells for over 1 year.^[^ ^14^ ^]^ Their inherent stability makes these devices especially useful for monitoring cardiac tissue function and identifying abnormal states. For example, we recently demonstrated a heart‐on‐a‐chip system that could monitor electrophysiological responses to acute hypoxia.^[^ ^15^ ^]^ In murine models, soft and bioresorbable MEAs implanted onto the heart were also shown to provide diagnostic readouts of organ‐level electrophysiology.^[^ ^16^ ^]^

Optogenetic techniques represent one promising path toward modulating cellular function with high spatial and temporal resolution and without the diffusion and kinetic limitations of drug‐based approaches. Microbial opsins have emerged as optically‐transduced modulators of transmembrane trafficking of ions and have been widely explored in neuroscience.^[^ ^17^, ^18^ ^]^ Opsins chimerized with G protein‐coupled receptors (GPCR, OptoXRs) were shown to change the intracellular concentration of secondary messengers thereby broadening the repertoire of optogenetic applications to non‐excitable cells. The potential of optogenetics has prompted advances in light‐mediated modulation of cellular function including vectors which target specific cell types or subcellular compartments, multi‐modal light sources for independent control over different optogenetic moieties, and fiber optic arrays to control tissue function in vivo.^[^ ^17^, ^18^ ^]^ Within the past decade these techniques have been extended to cardiac systems, with potential applications for arrythmia management and pacing, as a safer alternative to current pacemaker technologies.^[^ ^19^ ^]^

The activity of adenylyl cyclases (ACs), which convert adenosine triphosphate (ATP) into cyclic adenosine monophosphate (cAMP), has been a target for optogenetic modulation.^[^ ^20^ ^]^ Methods to regulate intracellular cAMP ([cAMP]_i_) using light would be relevant to a wide variety of cell types given the ubiquity of this second messenger. In the case of cardiomyocytes (CMs), cAMP phosphorylates protein kinase A (PKA) which in turn drives excitation‐contraction coupling^[^ ^21^ ^]^ involving L‐type Ca^2+^ channels, phospholamban, ryanodine receptors, and troponin I.^[^ ^22^ ^]^ In addition, cAMP stimulates hyperpolarization‐activated cyclic nucleotide‐gated (HCN) channels influencing the basal beating rate of CMs.^[^ ^23^, ^24^ ^]^ Rapid degradation of cAMP by phosphodiesterases^[^ ^25^ ^]^ also alters [cAMP]_i_ providing time‐limited control over cardiac contractility.

Optical modulation of [cAMP]_i_ in CMs may be achieved using photoactivatable ACs (PACs), which are native to microorganisms such as *Euglena gracilis*,^[^ ^26^ ^]^ *Oscillatoria acuminata*,^[^ ^27^ ^]^ and *Beggiatoa* (bPAC),^[^ ^28^, ^29^ ^]^ or engineered.^[^ ^30^, ^31^ ^]^ The small protein (350 aa) bPAC is a homodimer of an N‐terminal blue light using flavin (BLUF) photoreceptor domain and a C‐terminal type III AC domain.^[^ ^28^ ^]^ The flavin adenine dinucleotide needed for BLUF activity is readily available in animal cells. Activation of the AC site results from conformational changes in the BLUF domain brought about by exposure to blue light. Compared to other PACs, bPAC displays lower dark activity, up to 300‐fold light/dark activity, superior solubility, and short half‐life of the active lit state.^[^ ^30^, ^32^, ^33^ ^]^ We recently expressed bPAC in murine and human pancreatic *β*‐islet cells and found that photostimulation rapidly elevated [cAMP]_i_ with concomitant increase in insulin secretion.^[^ ^34^, ^35^ ^]^ Transplantation of bPAC‐expressing *β*‐cells into diabetic mice led to improved glucose tolerance and lowered hyperglycemia upon illumination.^[^ ^36^ ^]^

**Figure 1:**
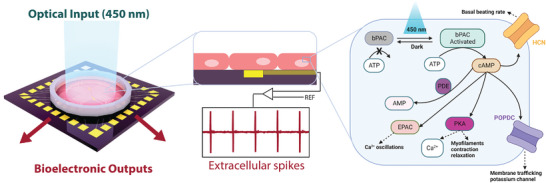
Overview of optical/bioelectronic control concept. (left) Bioelectronic chip featuring optical input and multiplexed bioelectronic outputs. (center) Detail of (red) CMs interfaced with (yellow) a single bioelectronic recording element, and representative bioelectronic outputs. (right) Proposed signaling pathway in bPAC‐transduced CMs whereby illumination stimulates cAMP synthesis and increases beating rate.

Herein we report an engineered cardiac system with two‐way communication capabilities enabled by a) time‐ and dose‐modulated blue light as an input, and b) multiplexed, real‐time bioelectronic recordings as outputs (**Figure** **1**). The bPAC was expressed for the first time in CMs to test our hypothesis that stimulation with light would elevate [cAMP]_i_ and the beating rate.^[^ ^37^ ^]^ We monitored CM activity via an integrated 32‐element MEA that provided continuous readouts in both light and dark states. The outputs allowed us to monitor the dynamic changes in beating in response to time‐limited and dose‐modulated periods of irradiation. Given the ubiquity of both MEAs and light sources (e.g., light‐emitting diodes) in bioelectronics, our integrated platform opens new avenues for closed‐loop control of function in cardiac or other tissues.

## 2 Results

### 2.1 AdbPAC Regulates [cAMP]_i_ Expression with Illumination

**Figure 2:**
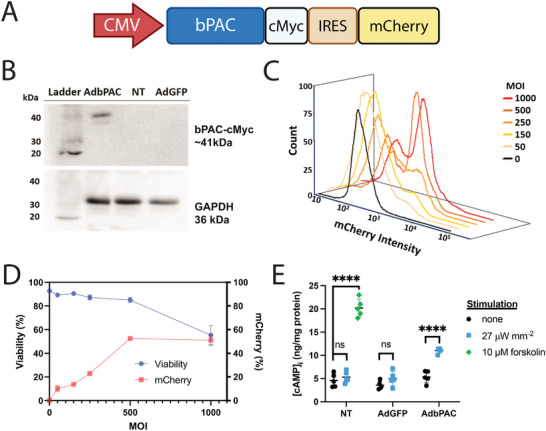
Optical interfacing using bPAC. A) Schematic of the bPAC cassette used for adenoviral transduction of CMs. B) Western blot analysis of samples from NT, GFP‐ and bPAC‐expressing CMs: Top panel: bPAC (ca. 41 kDa; detection of c‐Myc tag). Bottom: GAPDH (36 kDa; loading control). C) Flow cytometry for mCherry expression in CMs with MOI ranging between 0 and 1000. The black curve corresponding to MOI of 0 (NT), represents background fluorescence. D) Viability and mCherry expression in CMs 3 days post‐transduction with the same MOIs as in (C), *n* = 3. E) Intracellular cAMP expression in NT CMs or CMs transduced with AdGFP or AdbPAC and kept in dark (black), treated with 10 µm forskolin (green) or after 30‐min illumination (blue). Comparisons represent two‐tailed t‐test. Non‐stimulated groups have no significant difference by ANOVA. *n* = 5 for all groups, ^****^ *p* < 0.0001.

We first sought to determine whether expression of bPAC in CMs was possible. Primary rat CMs were transduced with an adenovirus^[^ ^34^ ^]^ (AdbPAC; **Figure** **2**A) carrying a cassette with i) the bPAC featuring a C‐terminal c‐Myc epitope for immunodetection, and ii) the fluorescent reporter mCherry for co‐expression, linked with an internal ribosomal entry (IRES) element. Western blot analysis of transduced CMs revealed a band at ca. 41 kDa, consistent with the expected size of bPAC (Figure 2B). No bPAC expression was observed in cells with no transduction (NT) or transduced with an adenovirus (AdGFP) carrying the green fluorescent protein (GFP) gene.

After successfully expressing bPAC in CMs, flow cytometry was employed to determine the fraction of bPAC^+^ cells in populations transduced with AdbPAC at various multiplicities of infection (MOI) (Figure 2C). The expression of mCherry was positively correlated with MOI over the range of 0–500 but plateaued between 500 and 1000 with expression at 52 ± 1.0% and 51 ± 4.2% at those two endpoints, respectively (Figure 2D). Increasing the MOI from 500 to 1000 also decreased the cell viability from 85.0 ± 2.1% to 55.3 ± 8.2%. For these and all subsequent experiments, MOI was set to 500.

Next, the modulation of [cAMP]_i_ was studied with light stimulation in bPAC‐expressing CMs (Figure 2E). The cells were illuminated with a 450 nm light emitting diode (LED) array continuously for 30 min. Here and for all subsequent experiments, unless noted otherwise, an intensity of 27 µW mm^−2^ was employed. Compared to bPAC^+^ CMs kept in the dark, irradiated cells exhibited nearly a twofold increase in [cAMP]_i_ (11.04 ± 0.49 vs 5.36 ± 1.15 ng cAMP/mg total protein, *p* < 0.0001, n = 5). By contrast, illumination did not significantly change [cAMP]_i_ in NT or GFP‐expressing CMs. In addition, no significant difference was observed between all three CM groups without exposure to blue light, supporting the hypothesis that bPAC has minimal dark activity and transduction alone does not alter [cAMP]_i_ expression. Treatment with 10 µm forskolin, an activator of AC,^[^ ^38^ ^]^ resulted in a 1.8 ‐fold greater [cAMP]_i_ in NT CMs compared to AdbPAC‐treated CMs stimulated with light (20.20 ± 1.92 vs 11.04 ± 0.49 ng cAMP mg^−1^ total protein, *p* < 0.0001, n = 5). This difference is consistent with observations in *Xenopus laevis* oocytes,^[^ ^39^ ^]^ and likely arises because forskolin enhances the activity of multiple native AC isoforms whereas light activates only bPAC.

### 2.2 Bioelectronic Recording Elements form Stable Interfaces with bPAC‐Expressing CMs

**Figure 3:**
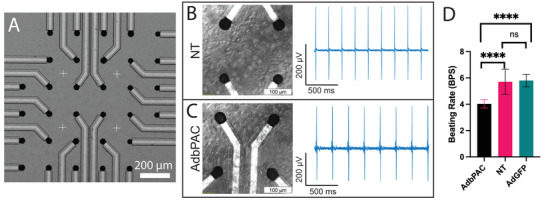
Bioelectronic interfaces with MEAs. A) Optical image of the MEA with 32 recording elements coated with platinum black. B,C) Representative bioelectronic interfaces with NT and bPAC‐expressing CMs: (left) Representative phase contrast images and (right) corresponding bioelectronic readouts at 7 DIV. D) Beat rate comparison between NT, AdbPAC‐, and AdGFP‐transduced groups at 6–7 DIV; *n* = 21, *n* = 9 and *n* = 5, respectively. ^****^ *p* < 0.0001 by one‐way ANOVA; error bars represent S.D.

We hypothesized that MEAs would stably interface with and record signals from CMs expressing bPAC. We fabricated our MEAs on silicon/silicon oxide substrates by photolithography.^[^ ^15^ ^]^ Each chip consisted of 32 Au‐pad recording elements, two large‐area quasi‐reference electrodes and associated interconnects. The recording elements had a pitch of 200 µm and the full array could interrogate a 1000 µm x 1000 µm region of cell monolayer (**Figure** **3**A). This area is sufficiently large to calculate wavefront propagation velocities, and to identify potential variations in intercellular signaling as we observed in hypoxia studies.^[^ ^15^ ^]^ Each chip was mounted onto a custom printed circuit board (PCB) and the cell culture area was defined by a silicone well adhered with poly(dimethyl siloxane) (PDMS) (Figure S1, Supporting Information). Recording elements were electrochemically coated with platinum black to increase signal‐to‐noise ratio by reducing impedance ca. 32x to 8.0 kΩ at 1 kHz (Figure S2, Supporting Information).

For all bioelectronics studies, CMs were plated onto MEAs coated with gelatin‐fibronectin to facilitate adhesion. The CMs formed confluent monolayers that began beating as soon as 24 h in vitro. Transduced groups were infected at 3 days in vitro (DIV) and maintained in standard culture conditions thereafter. At 6 DIV and beyond both NT‐ and AdbPAC‐transduced‐CM cultures continued to beat spontaneously and were unperturbed by the underlying devices (Figure 3B,C). Bioelectronic readouts demonstrated periodic beating with signal‐to‐noise which was similar to our previous studies on HL‐1 cell monolayers.^[^ ^15^, ^40^ ^]^

At 6–7 DIV we found that NT CMs beat rhythmically with a rate of 5.7 ± 1.0 beats per second (BPS) across nine distinct cultures, consistent with the range of values reported for similar cultures of primary CMs.^[^ ^41^ ^]^ Cardiomyocytes transduced with AdGFP beat at a statistically similar rate, 5.8 ± 0.5 BPS (p = 0.84), demonstrating that transduction did not significantly alter processes associated with contraction. Cells transduced with AdbPAC also beat rhythmically, but at a slower rate of 4.3 ± 0.6 BPS (compared to NT, *p* < 0.0001) (Figure 3D; Video S1, Supporting Information).

**Figure 4:**
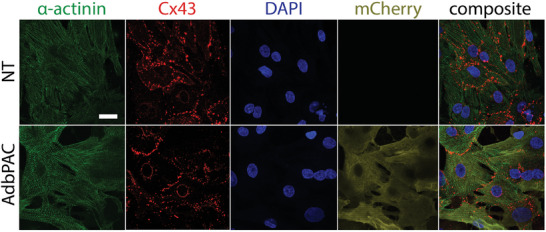
Expression of CM markers after illumination. Immunofluorescence images of NT and bPAC‐expressing CMs exhibiting cytoskeletal *α*‐actinin, Cx‐43 gap junctions, and mCherry. Nuclear DNA staining by DAPI is also shown. Prior to preparation for imaging, the cells were illuminated at 27 µW mm^−2^ for 30 min. Scale bar: 20 µm.

To further investigate the interface between cells and the chip surface we performed immunofluorescence studies on CMs cultured on glass substrates, after a 30‐min dose of illumination at 27 µW mm^−2^ (**Figure** **4**). Both NT and bPAC^+^ CMs exhibited features consistent with healthy cultures, including well‐defined nuclei, punctate gap junction expression at perinuclear and cell‐to‐cell adhesion sites based on staining of connexin‐43 (Cx43), and sarcomeres with well‐defined z‐disks (*α*‐actinin) characteristic of contractile CMs. In the case of CMs transduced with AdbPAC we also observed fluorescence throughout the cytosol, consistent with the expected distribution of the soluble mCherry tag.

### 2.3 Optical and Bioelectronic Interfaces Enable Two‐Way Communication with CMs

**Figure 5:**
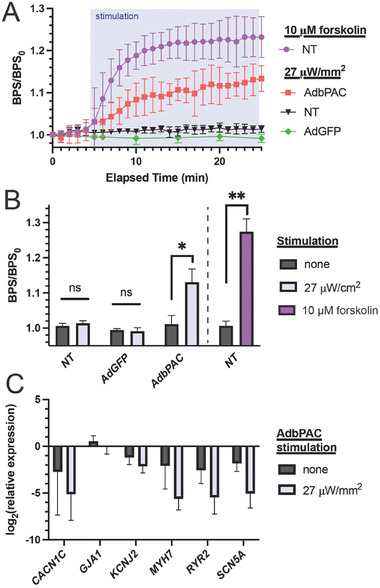
Response to illumination. A) Comparison of NT, bPAC‐ and GFP‐expressing CMs in response to light (*n* = 4 for each group) or forskolin (*n* = 3). B) Aggregate endpoint data from panel (A) at 25 min (photostimulation for 20 min). Comparisons are by paired two‐tailed *t*‐test, ^*^ *p* < 0.05, ^**^ *p* < 0.01. C) qPCR for key proteins associated with CM contractility. Light stimulation was applied for 30 min. Differences between groups are not significant (n.s.) by two‐tailed t‐test; *n* = 3. All error bars represent S.D.

With the bioelectronic system established, integrating the cellular and electronic components for proper interfacing, the signal readouts were used to evaluate the effect of photostimulation on CM function (**Figure** **5**A,B). For the first set of assays, we measured readouts from CMs in the dark state for 5 min, followed by illumination for 20 min. We abstracted the beating rate (beats per second, *BPS*) from the electrophysiological readouts and expressed it as the fold change to the initial, basal beating rate (*BPS/BPS_0_*), to account for variability between cultures. Upon irradiation we observed a progressive increase in beating rate in the AdbPAC group, reaching a 14% increase when illumination was terminated. In contrast, NT and AdGFP‐transduced CMs showed no statistically significant change over the same period. On the other hand, NT CMs treated with 10 µm forskolin increased their beating frequency by 23%. We note that the relative effects of forskolin and light stimulation on beating rates align with our observation of [cAMP]_i_ trends shown in Figure 2E.

The CMs were also examined by quantitative PCR for the expression of various markers relevant to contractility (*Gja1*, *Myh7*) and the handling of calcium (*Cacna1c*, *Ryr2*), potassium (*Kcnj2*) and sodium (*Scn5a*) channels (Figure 5C; Table S1, Supporting Information). The level of *Gja1* (encoding for Cx43), was not altered by the expression of bPAC and multi‐day, 30‐min illumination, which agrees with the immunofluorescence results (Figure 4). In addition, no significant suppression of other genes was noted in the AdbPAC cells kept in dark, suggesting that the functions of CMs remained unchanged with the expression of bPAC. Finally, we observed no statistical difference in the expression of any of the genes we examined between dark and irradiated groups. Taken together, these results demonstrate that key phenotypes associated with CM electromechanical behavior are unaffected by the transduction process or irradiation.

### 2.4 Irradiation Achieves Sustained Modulation While Preserving Normal Cellular Function

**Figure 6:**
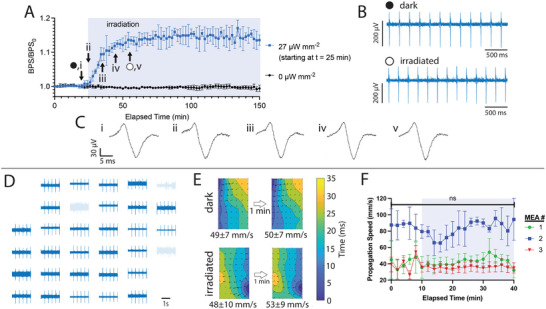
Electrophysiological analysis of optogenetically engineered CMs. A) Beating rate for chips without (black) or with (blue) 2 h irradiation starting at 30 min (light blue region); *n* = 3. B) Representative single‐device readouts in dark and light states corresponding to time points (⬤, ◯) noted in panel (A). C) Typical peak expansions at time points (i‐v) noted in panel (A). D) Representative readouts from an array of 32 recording elements in the dark state. E) Isochronal maps representing signal propagation (top) before and (bottom) during illumination. The area represented by each map is 600 µm wide x 1000 µm tall. F) Propagation speed versus time during dark and light (light blue region) states on three separate MEA cultures. Error bars represent S.E.M. among all velocity vectors on the isochronal map. Isochronal maps in panel (E) were calculated from data sets for MEA #1.

We next asked whether photoactivation of bPAC could maintain CMs at a stable, elevated beating rate. To this end, irradiation was applied for 120 min. The beating eventually plateaued at a 14% greater rate than the initial rate. Significantly, this increase reached 50% and 90% of maximum by *t_50_* = 7 and *t_90_* = 13 min, respectively. By contrast, the basal beating rate of bPAC‐expressing CMs in the dark state exhibited no more than 0.04% mean deviation from baseline (**Figure** **6**A).

While the characteristics of extracellular spikes are only weakly correlated with those of the action potential, their magnitude and shape are strongly dependent on the tightness of junctions between the CMs as well as the sheet resistance between the CMs and substrate.^[^ ^42^ ^]^ Unaveraged signals from a representative device (Figure 6B,C) demonstrate uniform shape and magnitude in both dark and light states. These data indicate that irradiation affects neither cell–cell coupling nor the CM adhesion to the substrate. Furthermore, the biphasic shape of these signals can be attributed to stimulus currents injected through gap junctions, followed by inward Na^+^ and Ca^2+^ currents.^[^ ^43^ ^]^ The stability of our signals is evidence that these ionic functions are not affected by irradiation.

To quantitatively assess signal conduction through the monolayer we considered multiplexed readouts from the MEA. Figure 6D shows signals from a typical chip with 29 out of 32 functional bioelectronic interfaces. In this case we used a subset of these devices in a 4 × 6 rectangular array, to construct isochronal maps, which provide information about wavefront propagation. We obtained readouts throughout a 10‐min dark period followed by 30 min of continuous irradiation. Representative maps from dark and irradiated states at 7 DIV showed uniform wavefront propagation patterns across the array. Despite long‐term drift, which is expected in the absence of pacing, these patterns were relatively constant over short (1‐min) timeframes (Figure 6E). Moreover, the average propagation speeds in light (49 ± 7.5 and 50 ± 7.2 mm s^−1^) and dark (48 ± 10 and 53 ± 9 mm s^−1^) states were not statistically different. These trends were observed across all time points and on three separate chips with CMs from different isolations (Figure 6F). While MEA #2 exhibited a greater propagation speed compared to the other two chips, this variation is to be expected since propagation speeds in neonatal rat cardiomyocyte cultures vary significantly over time (especially within the first two weeks^[^ ^44^ ^]^) and with the population of pacemaker cells present.^[^ ^45^ ^]^

Moreover, the propagation patterns observed here are consistent with those reported for healthy primary CM monolayers.^[^ ^46^ ^]^ Uniform signal propagation in space and time is indicative of proper and efficient electromechanical coupling, which is also essential for healthy cardiac function. In contrast, CMs in dysfunctional states presented significantly different propagation characteristics. For example, we previously reported hypoxic CMs with highly nonuniform propagation speeds with averages progressively decreasing over time, highly disordered isochronal maps indicative of multiple activation centers, and patterns changing significantly over ≤1 min time frames.^[^ ^15^ ^]^

### 2.5 Optical Modulation is Dose‐Dependent and Time‐Limited

**Figure 7:**
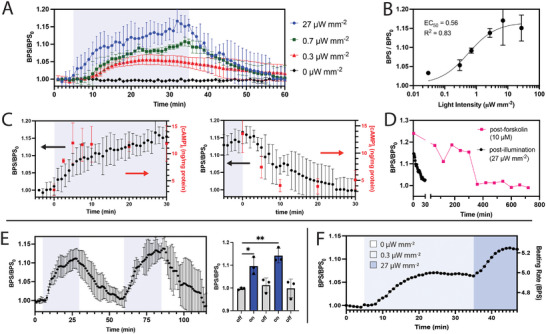
Modulation of contractile activity of CMs with time‐ and intensity‐dependent irradiation. A) Response to 30‐min illumination at selected intensities; *n* = 4 for 27 µW mm^−2^, *n* = 3 for other traces. B) Summary of beating rates following 30‐min irradiation: regression is agonist versus response curve. C) (black) Expansions of rising and falling edges of beating rates where *t* = 0 represents (left) turning the light on and (right) off. (red) [cAMP]_i_ measured under same conditions. D) Representative traces comparing post‐stimulation recovery after (magenta) 10 µm forskolin and (black) 27 µW mm^−2^ irradiation. E) (left) Response to two sequential 25‐min illumination cycles followed by 30‐min dark states; *n* = 3, and (right) corresponding summary statistics; one‐way ANOVA; ^*^ *p* < 0.05, ^**^ *p* < 0.01. F) Data are shown from a single device in response to stepwise irradiation at 0, 0.3, and 27 µW mm^−2^. All error bars represent S.D.

We hypothesized that the intensity and duration of illumination could be used to customize modulation of the contractile activity of CMs. To examine the relationship between illumination intensity and bPAC activity, we irradiated CMs for 30 min at intensities ranging from 0 to 27 µW mm^−2^ (**Figure** **7**A). After 30 min, 0, 0.03, 0.3, and 0.7 µW mm^−2^ irradiations yielded progressively larger values of *BPS/BPS_0_* but eventually plateaued at 2.7, 7, and 27 µW mm^−2^ regimens (p = 0.64). These results followed a standard agonist versus response curve^[^ ^47^ ^]^ with a half maximal effective concentration (*EC_50_*) of 0.56 µW mm^−2^ (Figure 7B). These responses track well with studies in *E. coli*, where [cAMP]_i_ versus light intensity followed Michaelis–Menten kinetics, with *K_m_* = 3.7 ± 0.4 µW mm^−2^.^[^ ^47^ ^]^

We next considered CM dynamics in response to step changes in irradiation. With a 27 µW mm^−2^ intensity, the beating rate leveled at *(BPS/BPS_0_)_100_* = 1.17 ± 0.03. The recorded beating rate closest to 50% of this plateau was *(BPS/BPS_0_)_50_* = 1.08 ± 0.04, at *t_50_* = 6 min (Figure 7C, left). These measurements are aligned with the independent experiments shown in Figure 6A, with *(BPS/BPS_0_)_100_* = 1.14 ± 0.02, *(BPS/BPS_0_)_50_* = 1.06 ± 0.1 and *t_50_* = 7 min. Under identical irradiation conditions, [cAMP]_i_ also increased until reaching a plateau, at 13.6 ± 3.5 ng mg^−1^ total protein, with *t_50_* < 2.5 min. We observed kinetics with similar trends upon turning off the light (Figure 7C, right): the elevated beating rate returned to baseline with *t_50_* = 6 min, while [cAMP]_i_ returned to its baseline of 4.2 ± 0.8 ng mg^−1^ total protein with *t_50_* < 5 min. Significantly, these light‐modulated CMs exhibited much faster off kinetics than CMs treated with 10 µM forskolin, which after three media washes remained at an elevated beating rate with *t_50_* > 5 h (Figure 7D).

By analogy to remotely‐triggered drug delivery systems,^[^ ^48^ ^]^ we hypothesized that time‐ and dose‐modulated illumination could be employed to achieve specific beating regimes. We first irradiated CMs over two cycles, with each cycle consisting of a 25‐min light phase followed by a 30‐min dark phase (Figure 7E). By the end of each of the two light‐on phases the CMs reached elevated *BPS/BPS_0_* of 1.10 ± 0.04 and 1.14 ± 0.03 (*p* < 0.05 and *p* < 0.01, compared to the preceding dark phase). There was no statistical difference between the three dark phases (p = 0.79) or the two light phases (p = 0.44). In a separate experiment, we irradiated CMs with a stepwise regime consisting of 0, 0.3, and 27 µW mm^−2^ for 5, 30, and 15 min, respectively (Figure 7F). In the representative single‐device example shown, we found that CMs responded in a correspondingly stepwise fashion with stable plateaus at 4.67, 5.00, and 5.29 BPS, respectively.

### 2.6 bPAC Expression is Stable over Multiple Days

**Figure 8:**
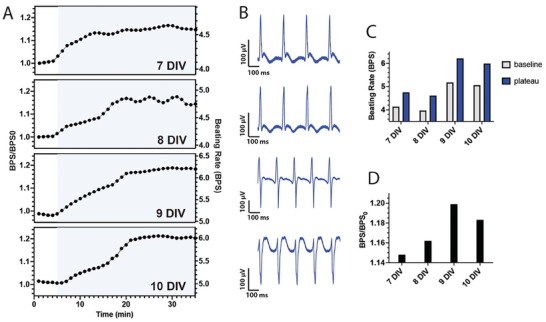
Multi‐day studies. A) *BPS/BPS_0_* and beating rate versus time for a single MEA irradiated on four consecutive days, 7–10 DIV. B) Illuminated‐state signals from a single, representative device on each day, corresponding to t = 25 min in Panel (A). C) Summary of beating rates at baseline and plateau regions. D) Summary of *BPS/BPS_0_* corresponding to beating rate data shown in panel (C).

To assess the stability of bPAC expression in CMs, we interrogated and irradiated the same MEA on four subsequent days (7–10 DIV), which is an experimental time course representative of other engineered cardiac tissue studies.^[^ ^10^ ^]^ Upon irradiation, the CM beating rate increased and eventually leveled off on all four days (**Figure** **8**A). Over this time period signals from a single, representative device transitioned from positive monophasic, to biphasic, to negative monophasic (Figure 8B), but on each day the signal‐to‐noise remained quite high (56, 48, 69, and 43 on DIV 7, 8, 9, and 10, respectively). Among other devices on the chip, we observed similar characteristics: signal amplitudes >64 µV, signal‐to‐noise >15 and root‐mean‐square noise <10 µV across all days and light and dark states. In addition, irradiation had little effect on these parameters between dark and light states (*p* > 0.01; Figure S3, Supporting Information) on any day. Over each of the four days we observed a generally increasing baseline beating rate (4.1, 4.0, 5.2 and 5.1 BPS; Figure 8C). This trend is consistent with bPAC‐negative CM cultures, which over time develop tighter intercellular junctions and greater contractility.^[^ ^10^, ^49^ ^]^ We also found that *BPS/BPS_0_* had a general upward trend of 1.15, 1.16, 1.20, and 1.18 (Figure 8D). Taken together, these results demonstrate the potential of optogenetic and bioelectronic interfaces for stable tissue output (contractility) regulation over extended time periods.

## 3 Conclusion

We demonstrated an integrated bioelectronic system permitting the modulation of the beating rate of CMs through photostimulation with concurrent monitoring of their response via multiplexed, bioelectronic readouts. CMs became amenable to altering their contractile activity with light by expressing bPAC where irradiation with blue light increased [cAMP]_i_ and subsequently the beating rate. Light‐based modulation of CM activity was accompanied by continuous measurement of the beating rate and thus the time‐dependent effects of photostimulation. We found that CMs could be maintained at a stable, elevated beating rate for at least 2 h. They responded quickly to step changes in irradiation, reaching a new steady state within 20–25 min. In addition, we found that we could achieve a desired beating rate by adjusting the light intensity. These responses were stable over at least four days.

While optical and electronic stimulation approaches have gained wide adoption in vitro and in vivo, their utility in the clinic has been limited because of the variability in responses across different cell types, between patients, and over time due to tissue plasticity. Bioelectronic therapies that incorporate both functional modulation and recording capabilities could address these shortcomings. We demonstrated a customized beating regime by modulating the timing and intensity of photostimulation (Figure 7E,F). While that system was open‐loop, relying on calibrated beating responses for our specific cell type, we note that both LED light sources and bioelectronic recording elements would be compatible with closed‐loop feedback architectures. Such platforms have been proposed for neuromodulation,^[^ ^50^ ^]^ and could readily be extended to cardiac and other systems. Our optical and bioelectronic interfaces are especially timely given recent advances in machine learning techniques, which are uniquely capable of interpreting large volumes of data to provide real‐time interventions, like in the detection and reversal of myocardial ischemia.^[^ ^51^ ^]^

Our modulation of bPAC activity allowed proper control of the CM contractile activity via the cAMP secondary messenger achieving the desired physiological effect. In response to irradiation we observed approximately a twofold increase in [cAMP]_i_ (Figure 2E), which is similar to the response induced by endogenous chronotropic agents such as catecholamines and glucagon.^[^ ^52^ ^]^ The commensurate increase in beating rate was 14% greater at steady state compared to the baseline. This elevation is also of clinical significance, as bradycardia (slow beating rate) is a condition with a heart rate less than 50–60 beats per minute; patients with heart rates in the range of 40–45 beats per minute have reported dizziness and other symptoms related to low blood flow.^[^ ^53^, ^54^, ^55^ ^]^

Moreover, the use of PACs to change CM beating may addresses some limitations of microbial opsins, which are leaky, lack the specificity of native ion channels, and are prone to desensitization after repetitive stimulation.^[^ ^56^ ^]^ Another advantage of our approach is that it leverages endogenous signaling pathways already in place and serving homeostasis, including those for CM excitation‐contraction coupling.^[^ ^57^, ^58^ ^]^ Additionally, bPAC‐induced cAMP‐based cellular responses may be faster and thus with physiologically more relevant kinetics compared to those of other optogenetic systems entailing gene transcription and translation steps for changes in response to transpire. The bPAC‐engineered CMs also exhibit a faster response with optostimulation compared to treatment with pharmacological agents. CMs maintained elevated beating rate after exposure to 10 µm forskolin and despite three media washes the t_50_ was longer than 5 h compared to min for bPAC^+^ CMs post illumination. This is most likely because the effects of forskolin are dependent on rates of diffusion and clearance.

Future versions of our technology might achieve a wider range of cardiomodulation through improved efficiency of gene delivery, the development of photosensitive analogs of other ACs that are present endogenously,^[^ ^37^ ^]^ and localization of PACs to specific domains such as plasma or Golgi membranes and transverse tubules, where subcellular cAMP pools activate distinct pathways.^[^ ^59^ ^]^ More broadly, cAMP localized to specific CM compartments is thought to exert preferential control over chronotropic (beating rate), inotropic (contraction force), lusiotropic (relaxation speed) and dromotropic (intercellular conduction velocity) effects. Subcellular localization of bPACs (as has been achieved with opsins^[^ ^60^ ^]^) could therefore afford exquisite control over multiple aspects of cardiac function, particularly if bPACs at distinct locations could be sensitized to and addressed by different wavelengths of light. This “whole cell” approach to cardiomodulation could be especially useful for investigating and treating dysfunctions such as atrial fibrillation,^[^ ^61^ ^]^ where there may be underlying biochemical deficits that are not addressed by conventional cardioversion therapies (e.g., pacemakers).

While this study explored cardiomodulation using a uniform irradiation source, multiplexed light sources such as arrays of LEDs could enable tunable, highly‐localized modulation of cardiac activity. Multisite or localized stimulation in conjunction with channelrhodopsin 2 (ChR2)‐expressing cardiomyocytes has been demonstrated in vivo^[^ ^62^ ^]^ to achieve cardiac pacing and in vitro to steer activation wavefronts^[^ ^63^ ^]^ and ablate spiral waves.^[^ ^64^ ^]^ This last point represents an important application of integrated MEA and stimulation technologies—i.e., the potential to identify an abnormality and provide a corrective response, in this case a stimulus applied to the core of the spiral wave to cause its dissolution. Localized perturbations could also enable mechanistic studies of intercellular cAMP trafficking and gradient PKA activity, which may arise endogenously from localized stimuli.^[^ ^65^ ^]^ With respect to varying the bPAC activity, future versions of our technology might incorporate multi‐functional sensing elements, with modalities to control beating rate, contraction force, relaxation speed, etc. Should chronic modulation cause phenotypic changes due to altered gene expression (as is the case with catecholamine‐induced stress response^[^ ^66^ ^]^), a closed‐loop feedback system could modify the simulation regime as necessary.

Effective bioelectronic therapies will require high‐density bioelectronic readouts with high reliability. Previously, we demonstrated stable recordings from planar chips and freestanding 3D scaffolds for at least 21 and 30 days,^[^ ^40^, ^67^ ^]^ respectively. Other groups have demonstrated stable recording elements^[^ ^13^ ^]^ and organic light‐emitting materials^[^ ^68^ ^]^ in vivo for 6–12 months. Ongoing efforts to tune the mechanical and chemical compatibility of bioelectronics are likely to further extend their lifetime and stabilize tissue integration.^[^ ^69^, ^70^ ^]^ In addition, recording artifacts due to optical stimulation (e.g., photoelectric effect) are much smaller and spatially confined compared to those from electrical stimulation^[^ ^71^ ^]^; in the case of our relatively low stimulation threshold (*EC_50_* = 0.56 µW mm^−2^, Figure 7B), these effects were negligible (Figure S3, Supporting Information, middle).

Clinical translation of any optogenetic or bioelectronic approach will require that the therapy be administered in a minimally invasive fashion. While the system reported here used a macroscale LED array and rigid bioelectronic device substrate, both technologies have also been demonstrated as microscale arrays on flexible and conformal substrates. Such systems could be implanted onto the surface of an organ, for example the epicardium after surgery.^[^ ^72^ ^]^ Very recently, mesh‐like bioelectronics were injected into the vascular system and implanted within the brain using an intracortical catheter, precluding invasive surgery altogether.^[^ ^73^, ^74^ ^]^ Additionally, PACs activated by red light (e.g., near infrared window light^[^ ^75^ ^]^), which exhibits deeper tissue penetration than blue light, could be employed with an extracorporeal light source, thereby eliminating the need for implanting microLED and reducing the implant size. Future versions of our optogenetic and bioelectronic platform could leverage these advances in materials science to achieve stable interfaces with the heart and other regions of the body to address a wide variety of medical conditions.

## 4 Experimental Section

### Chemicals and Reagents

All reagents used for cardiomyocyte isolation/culture and Pt black deposition were purchased from Sigma–Aldrich (St. Louis, MO): Horse serum, fetal bovine serum, penicillin‐streptomycin, aminocaproic acid, ascorbic acid, insulin‐transferrin‐selenium solution, collagenase II, forskolin, chloroplatinic acid, lead acetate. Four‐inch silicon wafers were purchased from University Wafer (South Boston, MA), and a two‐part Sylgard 184 PDMS kit was purchased from Dow Corning (Midland, MI).

### Cardiomyocyte Isolation

All animal procedures were performed in accordance with the Institutional Animal Care and Use Committee at Tufts University (Protocol #M2021‐110) and NIH Guide for the Care and Use of Laboratory Animals. Cardiomyocytes were isolated from P1 neonatal Sprague‐Daley rat pups (Charles River, Wilmington, MA; RRID:RGD_737891) as described.^[^ ^76^ ^]^ Briefly, the pups were sacrificed by conscious decapitation. Hearts were isolated and placed in ice‐cold PBS glucose solution to remove residual blood and connective tissue. Heart tissue was then minced into ≈1 mm^3^ pieces and suspended in 10 mL of PBS‐glucose solution. 7 mL of type II collagenase, warmed to 37 °C, was added to the tissue and placed on a tube rotator at 37 °C and 5% CO_2_. The tissue suspension was agitated to digest the collagen and release cardiomyocytes into the supernatant. After 7 min the tissue was gently titrated 5–6 times and allowed to settle in the conical tube for 3 min. The supernatant was then collected and added to 10 mL of DMEM and 10% fetal bovine serum (FBS) to inactivate the collagenase. 7 mL of fresh collagenase II solution was added to undigested tissue and this process was repeated until all the collagenase II had been used (seven steps in total). After the final digestion step, the cell solution was filtered through a 70 µm sieve into a new conical tube. The cells were then spun down at 100 xg for 5 min and resuspended in 30 mL of DMEM. To enrich the cardiomyocyte population, the resuspended cells were pre‐plated on tissue culture T‐175 flasks for 30–45 min. Unattached cells were removed and strained again with a 70 µm sieve and spun down at 100 xg for 5 min before cell counting and seeding.

### Cardiomyocyte Cell Culture

Prior to seeding, MEA chips were sprayed down with ethanol and irradiated overnight with UV light. To facilitate cell adhesion, chips were incubated at 37 °C with a solution of 1 mg mL^−1^ fibronectin dissolved in 0.02% gelatin solution for 1 h, after which they were ready for seeding. Myocardial culture media was prepared by adding 10% horse serum, 2% FBS, 1% penicillin‐streptomycin, 1% ITS solution, 6 mg mL^−1^ aminocaproic acid, and 50 µg mL^−1^ ascorbic acid into DMEM. Cardiomyocytes were seeded onto chips at 2.5 × 10^5^ cells cm^−2^, maintained in myocardial culture media, and incubated under standard conditions (37 °C, 5% CO_2_, 95% humidity) for 10 DIV. Unless stated otherwise, culture media was exchanged daily. Cultures were periodically screened for mycoplasma contamination using MycoAlert Mycoplasma Detection Kit (Lonza Bioscience, Morrisville NC).

### Generation of Adenovirus Carrying the bPAC Gene and Transduction

A human‐codon optimized bPAC gene was synthesized (GeneArt, ThermoFisher, Waltham, MA) from the available bPAC sequence (accession number: GU461306.2) and followed with a cMyc‐derived epitope, an IRES sequence and mCherry.^[^ ^34^ ^]^ The sequence of bPAC‐cMyc‐IRES‐mCherry was carried in pShuttle‐CMV vector for generation of adenovirus (AdbPAC) in 293AD cells with the AdEasy system (Agilent Technologies, Santa Clara, CA).^[^ ^34^ ^]^ The adenovirus carrying the GFP gene under the CMV promoter (AdGFP) was obtained from the Baylor College of Medicine Vector Development Core (Houston, TX). Purification and titer determination of the adenoviral particles were carried out using the Adeno‐X Maxi Purification Kit (Takara Bio USA, San Jose, CA) and QuickTiter Adenovirus Titer ELISA Kit (Cell Biolabs, San Diego, CA), respectively. The adenovirus propagation was conducted by transducing 293AD cells at 50% confluence with harvested when the cytopathic effect was complete. The cardiomyocytes were transduced at MOI as stated at 24 h after seeding. Following an incubation period of 48 h with viral particles, fresh medium was replenished, and assays were carried out at least 24 h after.

### Western Blot Analysis

Western blot analysis was carried out as reported.^[^ ^36^ ^]^ Briefly, cells were lysed in cell lysis buffer (Cell Signaling Technology, cat. no. 9803, Danvers, MA) supplemented with a protease inhibitor cocktail (Thermo Scientific, cat. no. 78425, Waltham, MA) and total protein was determined (ThermoFisher, cat. no. 23236, Waltham, MA). Cell lysates were boiled for 5 min at 95 °C, loaded to a polyacrylamide gel (30 µg of total protein/lane) and after gel electrophoresis and protein transfer to polyvinylidene difluoride membranes (Millipore, cat. no. IPVH00010, Burlington, MA). The membranes were blocked with 5% milk in Tris‐buffered saline with 0.1% Tween‐20 (TBST) for 1 h at room temperature. The membranes were incubated with primary antibodies against the c‐Myc epitope (Cell Signaling Technology, cat. no. 2278s, Danvers, MA) at 4 °C overnight, or with GAPDH (Sigma Aldrich, cat. no. G9545, St. Louis, MO) for 1 h at room temperature. Following 3 more TBST washes, secondary horseradish peroxidase (HRP)‐conjugated antibodies (Jackson ImmunoResearch Laboratories Inc., West Grove, PA) were added for 1 h at room temperature. Membranes were washed 3 times, developed with Clarity Max Western ECL Substrate (Biorad, cat. no. 1705062, Hercules, CA) and detected in a C‐DiGit blot scanner.

### Intracellular cAMP Measurements

Intracellular cAMP ([cAMP]_i_) was determined 72 h after transduction. One million cells/well were seeded in 12‐well plates and 5 wells were used for each condition. 1 h before the experiment, 1 mL of fresh medium was added to the wells. Forskolin at 10 µm (PeproTech Inc, Cranbury, NJ) was introduced at the start of the experiment. Cells from non‐treated, AdbPAC and AdGFP were incubated with or without illumination for various times at 37 °C and 5% CO_2_. Then, cells were lysed in 0.1 m HCl for determination of [cAMP]_i_ via enzyme‐linked immunosorbent assay (ELISA; Cayman Chemical Co., Ann Arbor, MI) following the manufacturer's instructions. The [cAMP]_i_ concentration was normalized by dividing with the total protein content measured by the Bradford method (ThermoFisher, Waltham, MA).

### Flow Cytometry

Harvested cardiomyocytes were treated with TrypLE Express Enzyme (1X) (Gibco, Waltham, MA) for 5 min and were centrifuged at 500x g for 5 min. The pellet was then resuspended in PBS and after a wash with PBS, cells were incubated with green fluorescent calcein‐AM dye (Invitrogen, Waltham, MA) for 20 min at room temperature. After the incubation and three washes with PBS, the stained cells were processed by an Attune NxT flow cytometer (Thermo Fisher Scientific, Waltham, MA) to measure calcein‐AM and mCherry expression. The results were analyzed with the FCS Express software (v. 6, De Novo Software, Pasadena, CA).

### Immunocytochemistry

Cardiomyocytes underwent immunostaining as described.^[^ ^77^ ^]^ Briefly, CMs were seeded on 35 mm glass‐bottom culture dishes (Matsunami Glass, Bellingham, WA) coated with fibronectin and gelatin, and were cultured and transduced as described above. The cells were fixed in 4% paraformaldehyde (Millipore‐Sigma, Burlington, MA) in PBS for 20 min and permeabilized with saponin (Millipore‐Sigma) in PBS for 1 h at room temperature in preparation for staining of nuclear antigens. Samples were washed three times (5 min each time) with PBS under light rotation and blocked with 5% BSA in PBS for 1 h. Antibodies against Connexin‐43 (rabbit; Abcam, cat. no. ab11370, Cambridge, MA), *α*‐actinin (mouse, Sigma, cat. no. A7811, St. Louis, MO), or mCherry (goat, OriGene, cat. no. TA150126, Rockville, MD) were added overnight at 4 °C in 1% BSA in PBS. After three washes with PBS, cells were then incubated with a corresponding secondary antibody conjugated at room temperature for 1 h (anti‐rabbit Alexa Fluor 488, anti‐mouse Alexa Fluor 647, anti‐goat Cy3, Jackson ImmunoResearch Inc., West Grove, PA) in 1% BSA in PBS. After three washes with PBS, nuclear DNA was stained with DAPI (Millipore‐Sigma, Waltham, MA) for 10 min. Following another three washes in PBS, VECTASHIELD Antifade Mounting Medium (Vectorlab, Newark, CA) was added to the coverslip. Samples were visualized with a Leica TCS SPE confocal microscope (Leica Microsystems, Wetzlar, Germany).

### RNA Extraction, RT‐PCR, and Quantitative PCR Analysis

Cardiomyocytes were isolated from three separate litters and plated on 6‐well plates for each condition. Total RNA was extracted using TRIzol (ThermoFisher, Waltham, MA, USA) according to manufacturer's instructions as reported.^[^ ^78^ ^]^ Reverse transcription was performed at 70 °C for 5 min and 42 °C for 60 min with 1 µg total RNA using ImProm‐II reverse transcriptase (Promega, Madison, WI, USA) and 250 ng oligo(dT)_12‐18_ primers (ThermoFisher, Waltham, MA, USA). The resulting complementary DNA (cDNA) and primers were mixed with PowerUp SYBR Green Master Mix (Applied Biosystems, Waltham, MA), and was analyzed on a StepOne Plus qPCR thermocycler (Applied Biosystems, Foster City, CA, USA) by quantitative PCR (qPCR) for 40 cycles at 58–60 °C annealing temperature depending on primer set. Primer sequences are listed in Table S1 (Supporting Information). Gene expression was analyzed with the ΔΔCT method.^[^ ^79^ ^]^ GAPDH served as the endogenous control of gene expression levels.

### MEA Fabrication

MEAs consisted of circular 30‐µm diameter gold recording elements and SU‐8 passivated interconnects fabricated on a silicon/silicon oxide substrate.^[^ ^15^, ^40^ ^]^ Briefly, photomasks were designed using AutoCAD (Autodesk Inc., San Rafael, CA). The device and interconnect layers were fabricated with LOR 3A and S1813 and metallized with 7 nm Cr/50 nm Au by sputter deposition (NSC‐3000, Nano‐Master, Inc., Austin, TX). Interconnects were passivated with 2 µm thick SU‐8 photoresist (SU‐8 2002, Kayakli Advanced Materials, Westborough, MA). MEA chips were mounted onto a custom printed circuit board (PCB) with 10‐pin surface mounted connectors (2.54 mm pitch). Electrical connections between the MEA and PCB were defined with silver epoxy (CW2400, Chemtronics, Kennesaw, GA). A 0.5‐in diameter silicone well (VWR, Radnor, PA) was adhered to the chips with PDMS which was cured at 65 °C for 4 h. The recording elements were electroplated with platinum black using aqueous chloroplatinic acid (1% w/v) and lead acetate (0.01% w/v) at −0.5 V for 15 s or total charge transfer of q = −100 nC using a Gamry 600+ electrochemical workstation (three electrode setup, Ag/AgCl reference electrode, Pt counter electrode).

### Impedance Measurements

Impedance spectra of recording elements were collected using an electrochemical workstation (Gamry Reference 600+, Gamry Instruments, Inc., Warminster, PA) with a three‐electrode setup (Ag/AgCl reference, Pt wire counter) and PBS solvent. It scanned between 10 and 10^6^ Hz with a perturbative potential of 20 mV.

### MEA Measurements

Measurements were performed between 6 and 10 DIV. Differential headstage amplifiers (891221, Harvard Apparatus, Holliston, MA) and adapters (890564, Harvard Apparatus, Holliston, MA) were mounted onto the pin connectors and data were collected using a 32‐channel recording system (USB‐ME32‐FAI, Multichannel Systems GmbH, Germany). Signals were amplified 1000x at the headstage, sampled at 25 kSa/s, then digitally filtered post‐hoc in MATLAB using a second order Type 1 Chebyshev filter with a 150 ‐2500 Hz bandpass. Filtered recordings with signal‐to‐noise <2 were excluded from subsequent analysis.

### Optical Stimulation

Cells were illuminated with 480 nm light from an LED panel light system (884667106091218, Resurs2 Corporation) mounted parallel to the of the chip at a fixed distance of 5.5 inches. An irradiation measurement probe (PM100D, Thorlabs Inc., Newton, NJ) was used to measure irradiance. For intensity variation experiments the light was attenuated with combinations of 1.0, 0.6, and 0.3 neutral density filters (53‐705, 53‐704, 53‐703, Edmund Optics, Barrington, NJ).

### Beating Rate and Isochronal Maps

Segments of filtered data (6 s length) were processed with custom scripts in MATLAB (MathWorks Inc., Natick, MA) as described.^[^ ^15^ ^]^ Beating rates and amplitudes were calculated by peak identification. *BPS_0_* represents the beating rate of the first segment in a time series and was used to find the fold change for all subsequent recordings in the series. Isochronal maps were generated by identifying relative time shifts among all functioning devices on the array. These activation times were interpolated and their gradient (∇ = i∂/∂x + j∂/∂y) was taken to generate a matrix of vectors representing the local slope of the isochronal map. Local propagation speeds were found by taking the inverse of each vector. Reported propagation speeds represent the average over all vectors on a single chip.

### Statistical Analysis

All statistical analyses were performed in Prism (v. 10, GraphPad Software, San Diego, CA). Groups were compared using a two‐tailed t‐test or ANOVA, and were presented as mean ± standard deviation, unless otherwise specified. A *p* < 0.05 was considered statistically significant.

## Conflict of Interest

The authors declare no conflict of interest.

## Supporting information

Supporting Information

Supplemental Video 1

## Data Availability

The data that support the findings of this study are available in the supplementary material of this article.
