# Supplementary material for: An Integrated Optogenetic and Bioelectronic Platform for Regulating Cardiomyocyte Function
Source: Adv Sci (Weinh). 2024 Jul 25;11(36):2402236. doi: 10.1002/advs.202402236 (PMC11423186; doi:10.1002/advs.202402236)
Supplement: Supplementary file 1 — Supporting Information [file ADVS-11-2402236-s002.pdf]

ADVANCED SCIENCE
Open Access

Supporting Information

for *Adv. Sci.*, DOI 10.1002/advs.202402236

An Integrated Optogenetic and Bioelectronic Platform for Regulating Cardiomyocyte Function

*Olurotimi A. Bolonduro, Zijing Chen, Corey P. Fucetola, Yan-Ru Lai, Megan Cote, Rofiat O. Kajola, Akshita A. Rao, Haitao Liu, Emmanuel S. Tzanakakis and Brian P. Timko**

Supporting Information

An Integrated Optogenetic and Bioelectronic Platform for Regulating Cardiomyocyte Function

*Olurotimi A. Bolonduro, Zijing Chen, Corey P. Fucetola, Yan-Ru Lai, Megan Cote, Rofiat O. Kajola, Akshita A. Rao, Haitao Liu, Emmanuel S. Tzanakakis, Brian P. Timko**

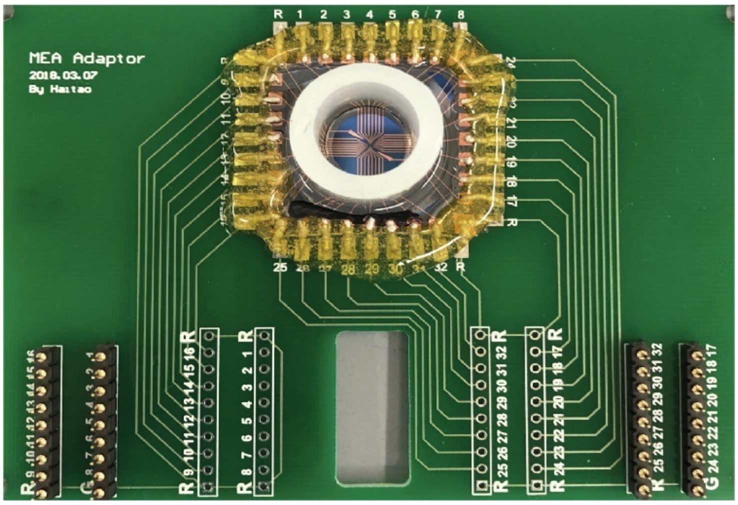

Figure S1. Assembly consisting of PCB, MEA chip and silicone well.

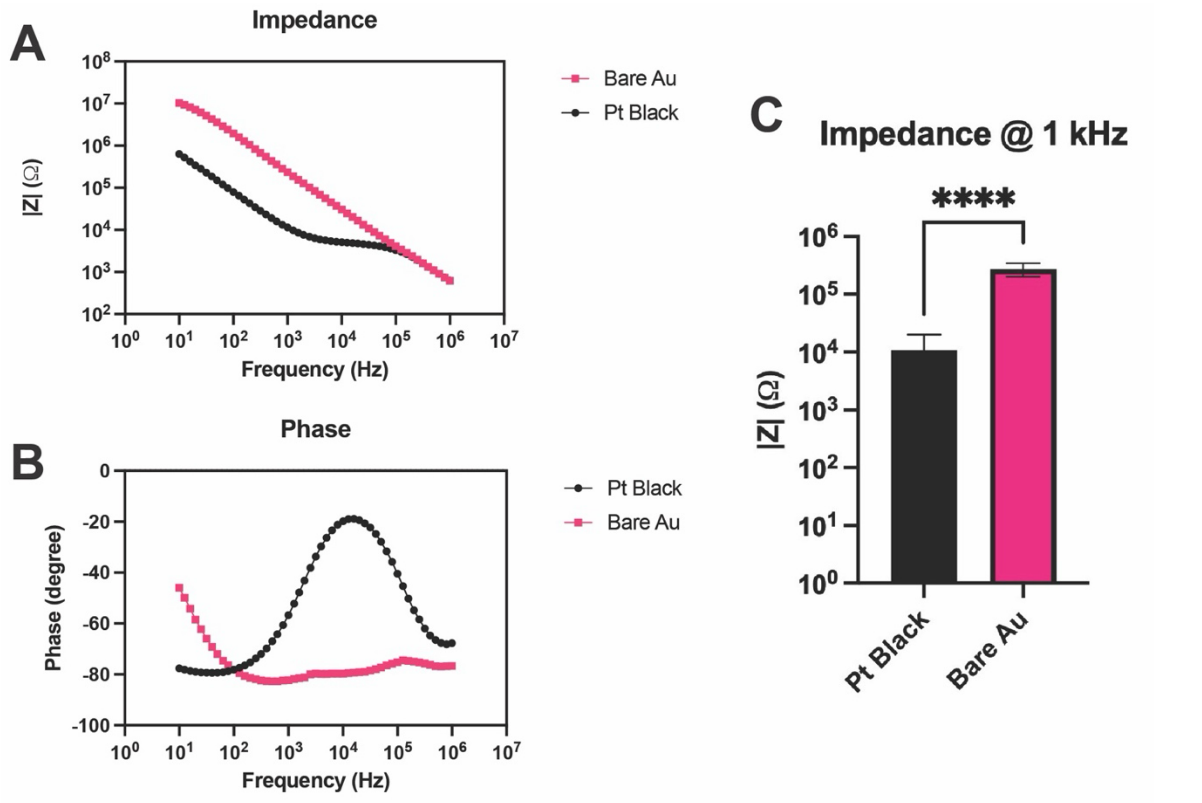

Figure S2. Chip impedance characteristics. (A,B) Impedance and phase spectra of (magenta) bare gold electrodes (black) Pt black-coated electrodes. (C) Impedance modulus at 1 kHz. n=20, **** p<0.0001.

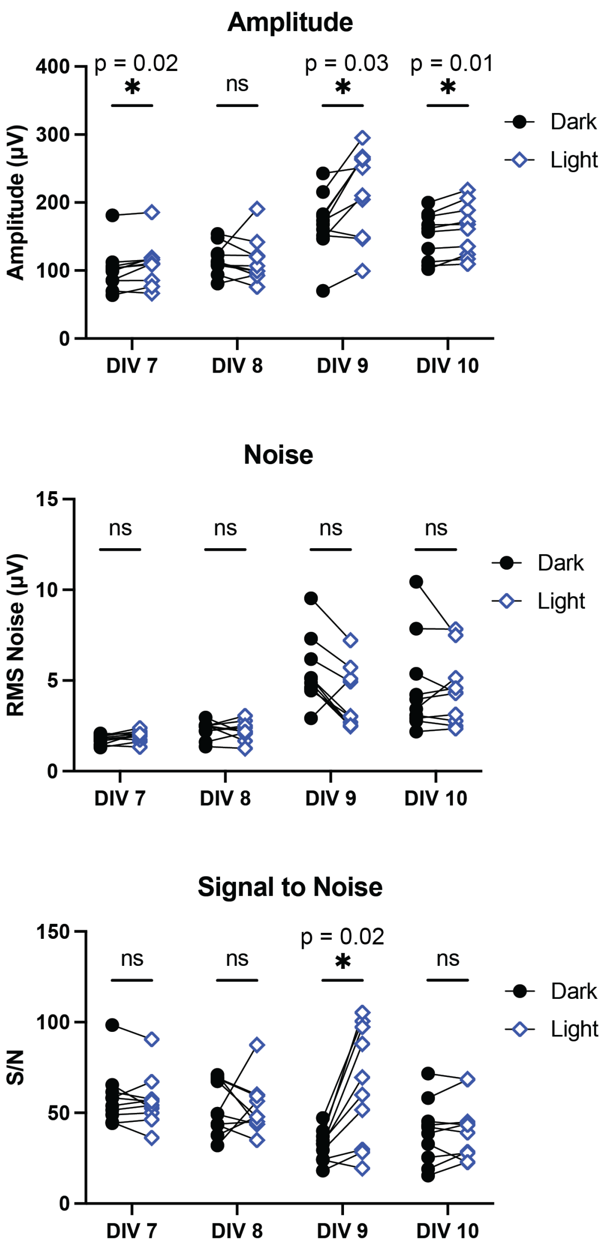

Figure S3. Multi-day statistics for individual devices. (top) Amplitude, (middle) root mean square baseline noise and (bottom) signal-to-noise for devices corresponding to the summary data shown in Figure 8, in dark (t=3) and light (t=25) states. Each data set represents the same devices. n=10, * $p<0.05$, Wilcoxon matched-pairs test with Holm-Šídák correction.

Table S1. Primers used for qPCR assays in this study (shown in a 5′-to-3′ orientation).

Gene	Amplicon Size (bp)	Primers	
Ryr2	99	Forward	ACTGCTGGGCTACGGCTAC
		Reverse	CTGAAGATGCGGAACCTCTC
Cacna1c	109	Forward	GTTGCCCTGGGTGTATTTTG
		Reverse	GGCTTTCTCCCTCTCTTTGG
Kcnj2	116	Forward	GCACAAGTACGGACTCACCT
		Reverse	TCCAAAGACAGAATCGGCCA
Gja1	99	Forward	CGCCGGCTTCACTTTCATTA
		Reverse	GGTGGAGTAGGCTTGGACCT
Myh7	145	Forward	TGGCACCGTGGACTACAATA
		Reverse	TACAGGTGCATCAGCTCCAG
Gapdh	92	Forward	GACATGCCGCCTGGAGAAAC
		Reverse	AGCCCAGGATGCCCTTTAGT
Scn5a	220	Forward	ACATGTTCAACTTCCAGACCTTC
		Reverse	ACGATGAGGAAGGAGATGATGAT
